# A retrospective study of the correlation between herpes zoster neuralgia and the serum neuron-specific enolase level in the largest dermatological hospital in Zhejiang province, China

**DOI:** 10.3389/fimmu.2022.972536

**Published:** 2022-10-06

**Authors:** Changyang Zhong, Ding Lin, Yuan Liu, Chunli Wu

**Affiliations:** ^1^ Cerebrovascular Department, Hangzhou Third People’s Hospital, Hangzhou, China; ^2^ Department of Cardiology, Hangzhou Third People’s Hospital, Hangzhou, China

**Keywords:** serum neuron-specific enolase, neuralgia, postherpetic, herpes zoster, elderly people

## Abstract

We studied the changes and clinical significance of the serum neuron-specific enolase (NSE) level in peripheral blood of patients with post-herpetic neuralgia (PHN).

**Material and methods:**

Patients with PHN were divided into a mild PHN group and a severe PHN group according to their scores on a 100-point Likert scale representing the degree of neuralgia. NSE levels in neuralgia patients and healthy volunteers were then compared by t-test. Receiver operating characteristic curve analysis was performed to evaluate the diagnostic efficiency of NSE for PHN. The correlation between NSE level and Likert scale score after treatment was analyzed.

**Results:**

NSE levels in PHN patients were higher than those in the healthy volunteers. Patients in the severe PHN group had higher NSE levels than those in the mild PHN group. NSE level at admission was associated with the Likert scale score recorded on the 14th day of treatment (136.1 ± 32.81 *vs*. 87.53 ± 16.23 pg/mL) (P < 0.05). The ROC curve of NSE levels for PHN is shown that the area was 0.8713 (95% confidence interval, 0.7861–0.9564; p < 0.0001).

**Conclusions:**

There was a correlation between NSE and PHN, in that the NSE level positively correlated with the short-term prognosis of patients with PHN.

## Background

The annual incidence rate of herpes zoster (HZ) is about 2–4.6 cases per 1,000 people in China, but it increases greatly to 9.1 cases per 1,000 people per year when considering just those 50–75 years of age (1). HZ is caused by the re-activation of latent varicella-zoster virus in the cranial nerve or dorsal root ganglia in adulthood (2). Patients with herpes zoster may experience persistent pain for several months after the acute phase as part of a phenomenon known as post-herpetic neuralgia (PHN), which is the most common chronic complication that may occur after herpes zoster and also the most common post-infection neuralgia (3). In recent years, studies have shown that the overall incidence rate of HZ in China is 6.64 cases per 1,000 people. Meanwhile, the incidence of PHN has been increasing, and there are about 4 million PHN patients in China (4). Even after the local skin lesions are healed, the local skin neuralgia can persist for months or years or even can develop into refractory neuralgia, which is often diagnosed in middle-aged and elderly people and causes long-lasting pain, especially in elderly women. Refractory neuralgia also leads to anorexia, insomnia, and depression, thus having a serious impact on patients’ lives (5).

Despite the harm brought on by PHN, the mechanism of the disease remains unclear in general. Currently, effective biomarkers to predict PHN are unknown, making it difficult to prevent or treat PHN. Some studies have reported that neuron-specific enolase (NSE) is not only related to small-cell lung cancer but also to nerve injury diseases (6–8). NSE is the γ-isoenzyme of enolase. Under normal circumstances, NSE exists primarily in the cytoplasm of neurons and neuroendocrine cells, with its recorded levels being highest in gray matter, followed by the spinal cord and peripheral ganglia (9). When neurons are damaged, the level of NSE *in vivo* rises. A few studies have shown that the serum NSE level significantly increases in patients with health conditions such as tumors, craniocerebral injuries, and central system infections (10, 11). However, there is no literature on the correlation between serum NSE levels and the degree of pain in HZ patients. Our study suggests that NSE can be used as a sensitive indicator to evaluate the severity and prognosis of PHN and has important clinical significance in this context.

## Material and methods

### Patients and eligibility criteria

All participants provided oral and written informed consent before their enrollment into the study. The design of this study conformed to the Declaration of Helsinki, and the study was approved by the Biomedical Ethics Committee of Hangzhou Third People’s Hospital. Forty patients (15 men and 25 women) with PHN seen at our hospital from April 2019 to October 2021 were selected as participants for this study. They ranged in age from 40–85 years old, with an average age of 68.3 ± 10.1 years. According to the diagnostic criteria of PHN, PHN in this study referred to pain at the rash site 1 month after HZ had been cured; thus, patients were required to meet this criterion for enrollment. We excluded patients who had a history of organic diseases, such as heart, lung, or kidney disease; patients with cognitive decline and language dysfunction; and patients with immune system disease, cancer, bacterial infections, or other relevant symptoms. Finally, patients also needed to be able to understand and accept their Likert scale score to be eligible to participate. In contrast, individuals <40 or >80 years of age, those with a recent history of immunosuppressant use, and pregnant and lactating women were excluded from this investigation.

A 100-point Likert scale (0 points, completely painless; 100 points, the most severe pain that may occur) is often used in clinical studies and case reports of PHN. Clinically, the minimum threshold score signifying meaningful PHN is 40 points; thus, clinically meaningful pain is confirmed when the Likert scale score is >40 points, while severe pain is confirmed when the Likert scale score is >70 points (12).

Study participants with PHN were divided into two groups according to their Likert scale scores. Patients with scores of >70 points were defined as having severe PHN (number = 20), and those with scores of >40 points were defined as having mild PHN (number = 20). Likert scale scores were calculated for the HZ neuralgia group on their first day of hospitalization.

### Detection of serum NSE levels and biochemical indicators

An initial total of 4 mL of venous blood was collected from each patient on the second day of admission before treatment, and another 4 mL of venous blood was obtained from each patient on the 14th day of treatment. All blood samples were put into anticoagulant tubes and sent to our hospital specimen bank for separation and cryopreservation. The serum NSE levels of test group and control group were determined by Roche Cobas E601 automatic electrochemiluminescence immunoanalyzer (Roche Diagnostic Products (Germany) Co., LTD.). The reagents were the supporting reagents of Roche Diagnostic Products (Shanghai) Co., LTD. Serum samples were collected using modular biochemical immunoassay analyzer (Roche Diagnostics, Basel, Switzerland) to detect Biochemical indicators, and the kit was manufactured by Hangzhou Novartis Bioengineering Co, Ltd. Biochemical indicators, including triglyceride, total cholesterol, tumor necrosis factor α, low-density lipoprotein cholesterol, and interleukin-10 concentrations. The condition of patients after treatment was assessed using the aforementioned Likert scale. The correlation between the NSE level at admission and the Likert scale score recorded after 14 days of treatment was also analyzed.

### Clinical data collection

Detailed medical history-taking, medication history-taking, and physical examinations were completed for all patients and healthy volunteers. Clinical data included age, gender, smoking history, drinking history, and blood pressure.

### Statistical analysis

Statistical analyses were performed using SPSS version 21.0 (International Business Machines Corp., Armonk, NY, USA). Patient characteristics were described with frequencies (percentages) or mean ± standard deviation values. Significant differences between groups were assessed by one-way analysis of variance. The chi-squared test was used for counting data. The diagnostic efficiency was calculated by receiver operating characteristic (ROC) curve analysis, and p < 0.05 was considered to indicate statistical significance. Figures were drawn using the SPSS Statistics international Business Machines Corp., Armonk, NY, USA.) and GraphPad Prism version 8.0 (GraphPad Software, San Diego, CA, USA) software programs.

## Results

A total of 40 patients with PHN and 40 healthy volunteers were enrolled in this study. The characteristics of the included patients and volunteers are shown in **Table 1**.

**Table 1:** The detailed clinical information of patients in postherpetic neuralgia and healthy volunteers.

Characters	mild postherpetic neuralgia (n = 20)	Severe postherpetic neuralgia (n = 20)	Healthy volunteers (n = 40)
Gender			
male	7	8	16
female	13	12	24
Age	68.3 ± 10.1	70.3 ± 8.4	69.5 ± 9.2
Likertscale score	44.5 ± 12.3	78.2 ± 7.3	/
Drinking	12	14	20
Smoking	16	15	24
Hypertension	14	18	/
Diabetes	7	10	/
TC(mmol/L)	4.52 ± 1.21	4.69 ± 0.69	4.01 ± 1.20
IL10(pg/mL)	4.03 ± 1.14	4.12 ± 1.22	4.01 ± 1.14
TG(mmol/L)	2.21 ± 1.26	2.47 ± 1.04	1.58 ± 0.97
TNF-α(pg/mL)	7.11 ± 1.11	7.41 ± 1.24	7.03 ± 1.12
LDL-C(mmol/L)	3.01 ± 1.18	3.95 ± 1.09	2.82 ± 1.07

TG, Triglyceride; TC, Total Cholesterol; LDL-C, Low Density Lipoprotein Cholesterol.

IL10, interleukin; -10, TNF-α: tumor necrosis factor-α. (Mean ± SD).

There were no marked differences between patients with PHN and volunteers in variables, including in age, gender, tumor necrosis factor α, total cholesterol, triglyceride, interleukin-10, or low-density lipoprotein cholesterol (all *P* > 0.05).

**Figure 1:**
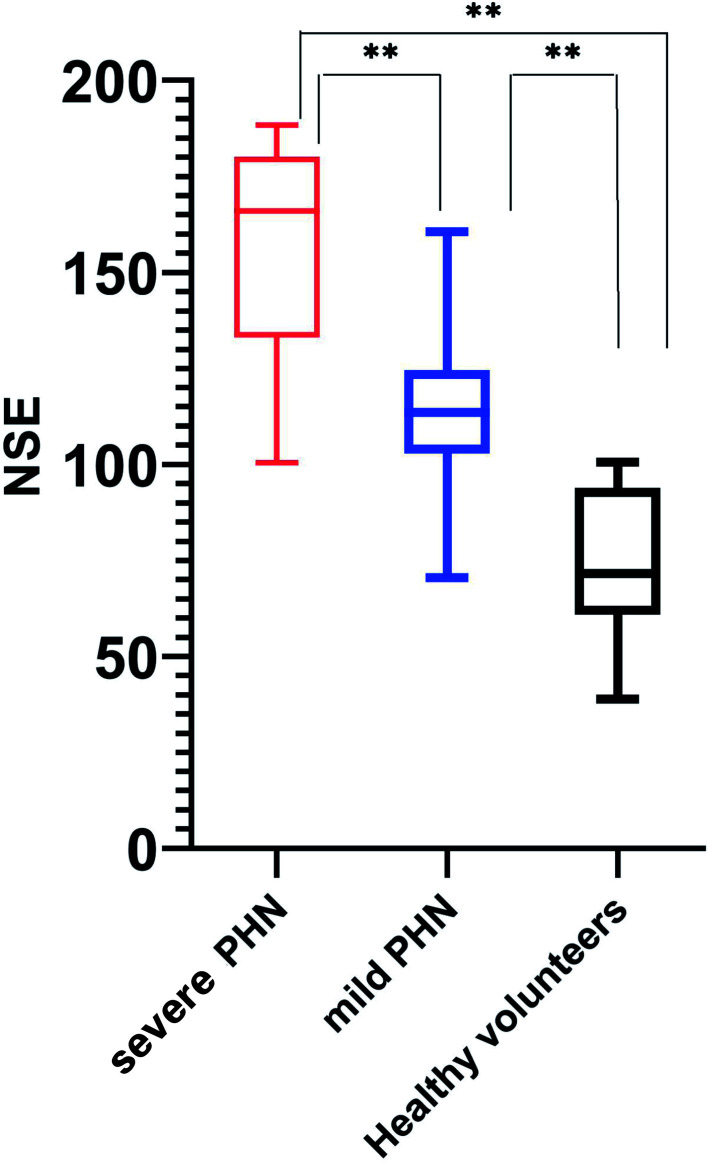
Serum NSE levels in all groups. ** means that the p-value is much less than 0.

There was a significant difference in NSE levels between the mild and severe PHN groups, with patients in the severe PHN group having higher NSE levels (156.6 ± 28.01 *vs*. 113.6 ± 18.91 pg/mL, p < 0.05). All patients with PHN, whether mild (156.6 ± 28.01 pg/mL) or severe (113.6 ± 18.91 pg/mL), had higher NSE levels than healthy volunteers (74.79 ± 20.02 pg/mL, p < 0.05) (**Figure 1**).

**Figure 2:**
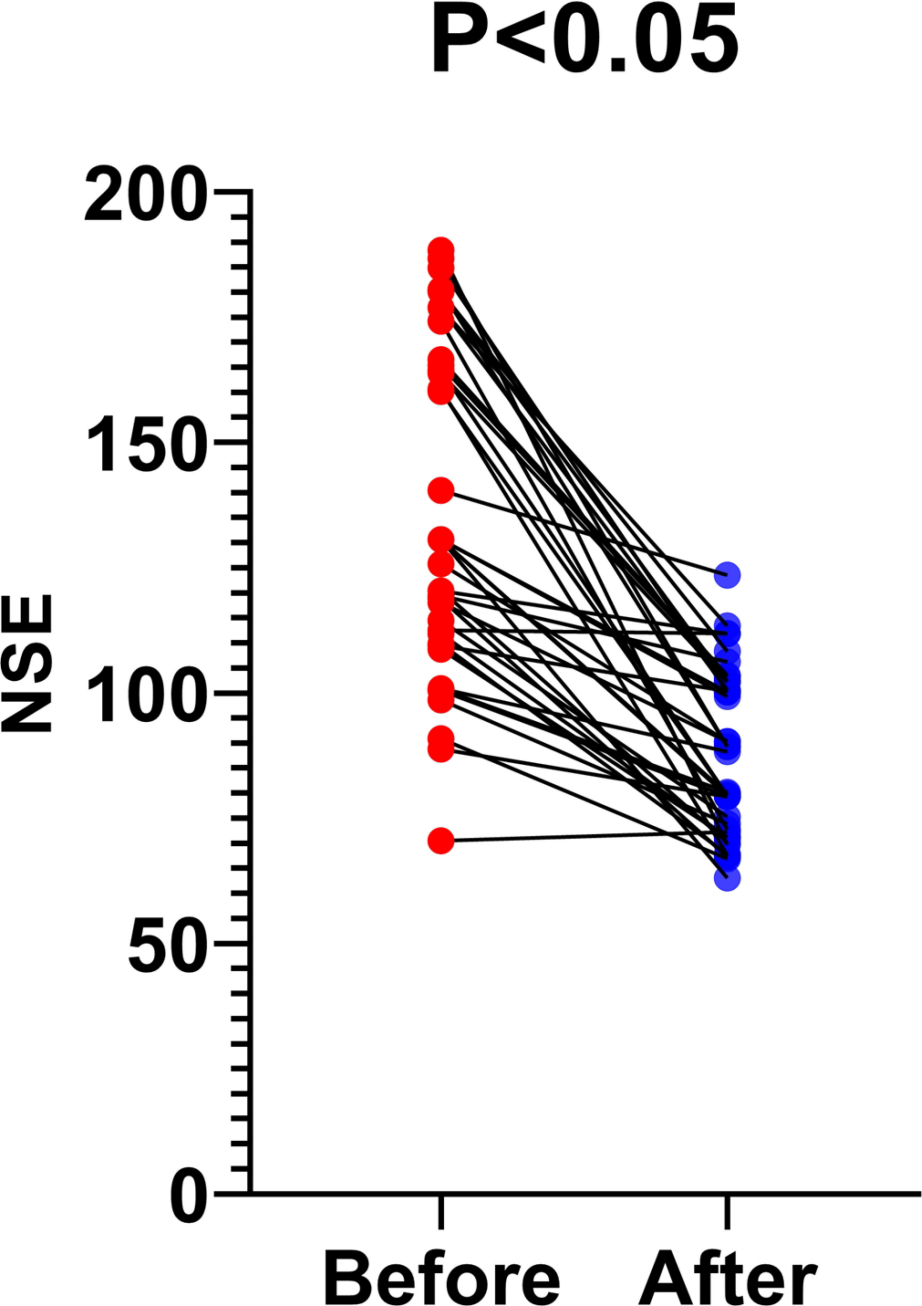
Changes in serum NSE levels after treatment for herpes zoster neuralgia.

Forty patients had herpes zoster neuralgia. All patients were administered appropriate doses of oral gabapentin and an intramuscular injection of 1,000 μg of mecobalamin daily. Patients with severe pain were given 5mg oxycodone hydrochloride oral sustained-release tablets once a day, and patients with severe pain were given the same dose again at 8-hour intervals. NSE levels decreased in this population significantly after 14 days of treatment (136.1 ± 32.81 *vs*. 87.53 ± 16.23 pg/mL). Changes in NSE levels before and after treatment were statistically significant, as shown in **Figure 2**.

**Figure 3:**
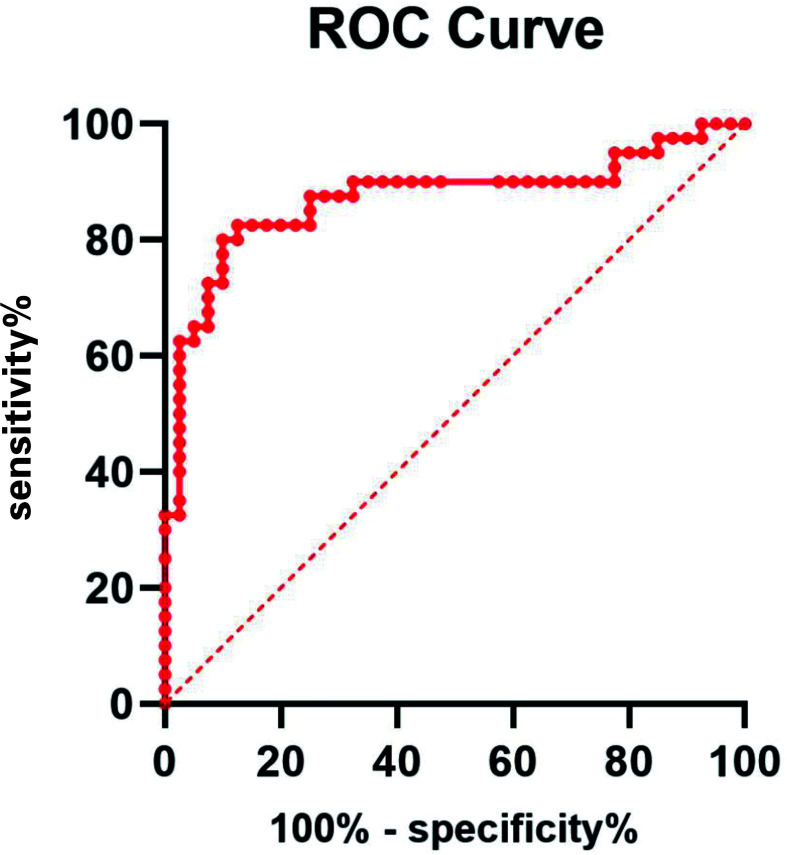
ROC curve of NSE level for PHN. The area under the ROC curve value was 0.8713 (95% confidence interval, 0.7861–0.9564; *P* < 0.0001).

The ROC curve of NSE levels for PHN is shown in **Figure 3**. The area under the ROC curve value was 0.8713 (95% confidence interval, 0.7861–0.9564; p < 0.0001). According to the ROC curve, the cutoff value of the NSE level for PHN was 104.8 pg/mL, with 82.5% sensitivity and 82.5% specificity, as shown in **Figure 3**.

After the 14th day of treatment, all patients with PHN were assessed using the Likert scale, and their results are shown in **Table 2**. NSE levels at admission were associated with Likert scale scores recorded on the 14th day of treatment (*P* < 0.05).

**Table 2:** Before and after 14 days of treatment, serum NSE levels in each group were correlated with the Likert Scale score.

Likert scale score (point)	0-40		40-70	≥70	P
**On admission**					
Patient (case)		20		20	
Serum NSE level (pg/mL)	74.79 ± 20.02	113.6 ± 18.91		156.6 ± 28.01	<0.05
**14 days after treatment**					
Patient (case)	18	17		5	
SerumNSE level(pg/mL)	73.18 ± 21.13	114.32 ± 18.72		157.01 ± 27.02	<0.05

Patients with a Likert scale score of more than 70 points were defined as having severe PHN.

Patients with a Likert scale of more than 40 points were defined as having mild PHN.

## Discussion

In our study, it was found that the serum NSE level positively correlated with the Likert scale score, suggesting that NSE plays a role in the pathological progression of PHN. Specifically, the correlation suggested that the higher the NSE level, the more pain patients can be expected to feel and the more serious their nerve injuries are, which further confirms that NSE is related to nerve injury. Sun et al. previously showed that the NSE level is related to nerve injury (13), consistent with our study. On this basis, our research further reveals that the serum NSE level in patients with severe PHN was significantly higher than that in patients with mild PHN or the healthy control group, suggesting that HZ patients with high NSE levels are more likely to have severe PHN. This finding provides a basis for understanding the disease evolution of HZ patients and for early diagnosis of severe PHN, and it provides a reference for adjusting the early treatment plan for HZ. Early intervention and regular treatment of patients with elevated NSE levels could help to alleviate their pain and improve their quality of life.

In our study, the NSE level was statistically significantly different in HZ patients before and after treatment. In addition, we found that the NSE level in patients with PHN at admission correlated with the Likert scale score recorded on the 14th day after diagnosis. Knysh et al. studied a number of molecules and mentioned that the level of NSE correlated with PHN, consistent with our research, suggesting that NSE might be involved in the progression of herpetic neuralgia (14). Consistent results have also been confirmed in eastern Asian and northern European populations. However, it had not been shown before that the NSE level is closely related to PHN prognosis. Our results suggest that with the improvement of PHN, the NSE level will decrease, and pain symptoms will improve, indicating that the NSE level can effectively reflect the curative effect of PHN treatment. Thus, we think that NSE levels in patients with PHN at admission may be a predictive factor for short-term prognosis. Our study can provide a basis for judging the therapeutic effect of PHN.

In our study, NSE may be a diagnostic indicator of PHN with 85% sensitivity and 87.5% specificity. The possibility of herpes zoster developing into herpes zoster neuralgia can be judged early by the level of NSE.

Importantly, serum NSE is affected by many factors. When specimens are hemolyzed, the level of NSE may increase. NSE levels are also higher in patients with brain disease, small-cell lung cancer, thyroid cancer, and neuroblastoma. Non–herpes zoster–related factors, including the above factors, should be strictly excluded. In addition, pain is subjective. Some mental states, such as anxiety and depression, greatly influence an individual’s perception of their pain, and pain tolerance can vary between people. Therefore, pain scores can be affected by different individual factors, so it is often necessary to include spiral computed tomography imaging, magnetic resonance imaging, cerebrospinal fluid, and a histopathologic biopsy in the evaluation of nerve injury. Future research should expand the sample size, make the sample more heterogeneous, and follow PHN patients for >6 months.

## Conclusions

The level of NSE can reflect the degree of nerve injury, which is of great significance in judging the prognosis of patients with herpes zoster and intervening early to prevent the occurrence of neuralgia.

## Data availability statement

The data analyzed in this study is subject to the following licenses/restrictions: The data that support the findings of this study are available from the corresponding author upon reasonable request. Requests to access these datasets should be directed to zhonghening@163.com.

## Ethics statement

The study was approved by the Biomedical Ethics Committee of Hangzhou Third People’s Hospital. The patients/participants provided their written informed consent to participate in this study.

## Author contributions

CZ: writing - original draft, conceptualization; DL: writing - review and editing, validation; YL: conceptualization and formal analysis; CW: validation and data curation. All authors contributed to the article and approved the submitted version.

## Funding

This project was supported by the Medical and Health Science Technology Project, Hangzhou, Zhejiang, China [grant number 2022KY977].

## Conflict of interest

The authors declare that the research was conducted in the absence of any commercial or financial relationships that could be construed as a potential conflict of interest.

## Publisher’s note

All claims expressed in this article are solely those of the authors and do not necessarily represent those of their affiliated organizations, or those of the publisher, the editors and the reviewers. Any product that may be evaluated in this article, or claim that may be made by its manufacturer, is not guaranteed or endorsed by the publisher.
